# Evaluation of at-risk infant care: comparison between models of primary health care

**DOI:** 10.11606/s1518-8787.2019053001063

**Published:** 2019-11-12

**Authors:** Alessandra Giannella Samelli, Gislene Andrade Tomazelli, Maria Helena Morgani de Almeida, Fátima Corrêa Oliver, Silmara Rondon-Melo, Daniela Regina Molini-Avejonas

**Affiliations:** I Universidade de São Paulo. Faculdade de Medicina. Curso de Fonoaudiologia do Departamento de Fisioterapia, Fonoaudiologia e Terapia Ocupacional. São Paulo, SP, Brasil; II Universidade de São Paulo. Faculdade de Medicina. Curso de Terapia Ocupacional do Departamento de Fisioterapia, Fonoaudiologia e Terapia Ocupacional. São Paulo, SP, Brasil

**Keywords:** Infant, Newborn, Risk Groups, Primary Health Care, Health Care Quality, Access, and Evaluation, Family Health Strategy, Unified Health System

## Abstract

**OBJECTIVES:**

To analyze the health care network for at-risk infants in the western region of the city of São Paulo, with the primary health care as coordinator, and to compare the presence and extension of attributes of primary health care in the services provided, according to the service management model (Family Health Strategy and traditional basic health units).

**METHODS:**

A survey was conducted with all at-risk infants born in the western region of São Paulo between 2013 and 2014. The children were then actively searched for a later application of the PCATool – child version. The total of 233 children were located in the territory; 113 guardians agreed to participate, and 81 composed the final sample.

**RESULTS:**

Regarding the results of PCATool for overall and essential scores, the units with Family Health Strategy were better evaluated by users, when compared with traditional basic health units, showing a statistically significant difference. However, these scores were low for both management models. Regarding attributes, the Family Health Strategy presented better performance compared with traditional basic health units for most of them, except for coordination of information systems. Of ten assessed attributes, seven reached values ≥6.6 for Family Health Strategy and two for the traditional basic health unit.

**CONCLUSIONS:**

Regardless of the type of management model, low overall and essential scores were found, indicating that guardians of at-risk infants rated some attributes as unsatisfactory, with emphasis on accessibility, integrality and family guidance. Such a performance may have negative consequences for the quality and integrality of these infants’ health care.

## INTRODUCTION

Health Care Networks (HCN) are sets of health services linked together with common mission and goals. They offer continuous and comprehensive care to a given population, coordinated by the primary health care (PHC), composing functional health systems^1^ . In this perspective, the Brazilian Ministry of Health (MH) has defined four thematic networks: *Cegonha* (Stork Network), Urgency and emergency care, Psychosocial care, and Healthcare for people with special needs^2^ . The Stork Network was implemented to ensure humane care to pregnant women and full monitoring of the child’s first two years of life. This project must ensure the binding of the mother-infant pair, promote comprehensive health care quality, and conduct active search for children in vulnerable situations.

The Family Health Strategy (FHS) was established to change the traditional way of health care provision, aiming to promote a model with PHC in care coordination, respecting principles of family integrity and community relationships, universal service and equity^3 , 4^ . Although previous studies indicate greater user satisfaction with the FHS, it is still necessary to expand assessments about management and provision of services and care, using standardized and validated questionnaires^3^ . MH has been developing strategies for assessment and monitoring of PHC, also considering the satisfaction or perception of professionals and users. This type of assessment process guides the decision-making process on services, aiming to transform PHC in gateway, with the reliability and resolution expected for a quality healthcare network^5^ .

The Primary Care Assessment Tool (PCATool) was created based on an assessment model of health quality services, measuring aspects of structure, process, and results^6^ . The PCATool aims to assess essential attributes and derivatives of PHC in services to adults and children, relating user experiences with professionals and health service, measuring their satisfaction. The instrument was translated, adapted and validated into Brazilian Portuguese and is used to assess PHC^7 , 8^ .

This study aimed to analyze the healthcare network for at-risk infants in the western region of São Paulo, with PHC as care ordinator. As specific objectives, the objective was to compare the presence and extent of PHC attributes in services provided for at-risk infants of different management models (FHS and traditional).

The hypotheses of this study include:

1. The basic health units (BHU) with FHS will be better assessed when compared with the traditional BHU model (tBHU), and scores will be above 6.6.
2. When considering each attribute individually, FHS will present a better performance, particularly in the attributes: first contact access, accessibility, longitudinality, coordination of care integration, and family and community orientation.

## METHODS

Descriptive cross-sectional study approved by the Ethics Committees of the University of São Paulo Medical School (FMUSP) (189/14) and the Municipality of São Paulo (32273014.8.3001.0086). Children’s guardians signed the informed consent form.

The search was conducted seeking all newborns classified as high-risk infants in the western region of São Paulo, Brazil (coordinated by the Technical Healthcare Supervision of Butantã – STS/BT), between August 1, 2013 and February 28, 2014 by certificate of live birth (CLB).

The criteria to classify at-risk infants were those determined by Decree No. 43.407^9^ , once the subjects belonged to STS/BT, which had a specific demand regarding the territorial diagnosis characterization of these infants. Risk criteria adopted by STS/BT are: birth weight ≤ 2,500 g, Apgar one minute after birth ≤ five and maternal age ≤ 16 years old. To be included in the study, the infant should meet at least one of these risk criteria. Thus, there were some differences as to the risks described in the MH booklet^10^ .

**Figure 1:**
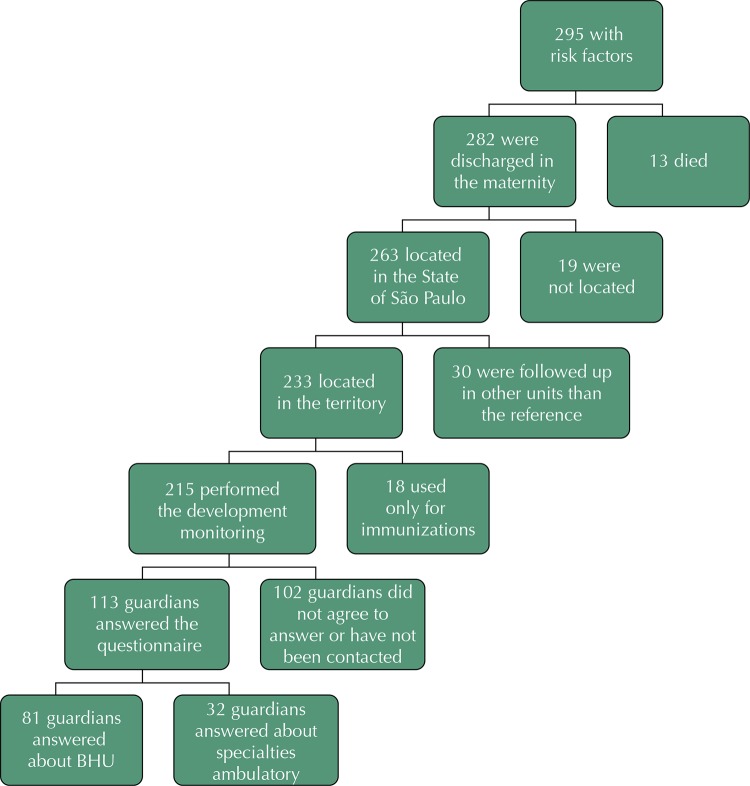
Flowchart for selection of study participants.

STS/BT has a population estimated at 442,198 dwellers, being 31,924 children between 0 and 4 years. It also has 14 BHU, seven FHS and seven tBHU, a specialty outpatient clinic and two maternity hospitals as reference for at-risk infants who were born/reside in the territory. Target population consisted of the guardians of at-risk infants in this region, registered in one BHU and selected as shown in Figure 1 .

Only data concerning to PHC assessment were included; thus, 32 subjects who responded to PCATool referring to the secondary care were excluded. Therefore, the final sample was composed of 81 participants:

First, we applied the socio-economic survey of the Brazilian Association of Research Companies^11^ . Then we applied the PCATool – child version^6^ , which assesses the following key attributes of PCH: first contact access (access and use of services); longitudinality (regular source of care and use over time); integrality (services available and focused on comprehensive care, including referral to other services), and care coordination (continuity and recognition of problems treated in other services, as well as care integration). The derivative attributes assessed qualify the actions of services: family and community orientation^6^ .

The assumption for each normal variable distribution was assessed using the Shapiro-Wilk test. Descriptive analyses of the numerical variables were displayed as means, medians and interquartile ranges (IQR); categorical variables by percentages.

As hypothesis tests, we used: Mann-Whitney (numeric variables), chi-square or Fisher’s exact test (categorical variables), assuming a significance level of p ≤ 0.05. Analyses were performed in SPSS 21 software (Windows).

## RESULTS

Of the 81 infants involved, 44 were boys (54.32%) and 37 were girls (45.67%). Regarding the management model, 42 infants (51,85%) were linked to FHS and 39 (48,15%), to tBHU.

**Figure 2:**
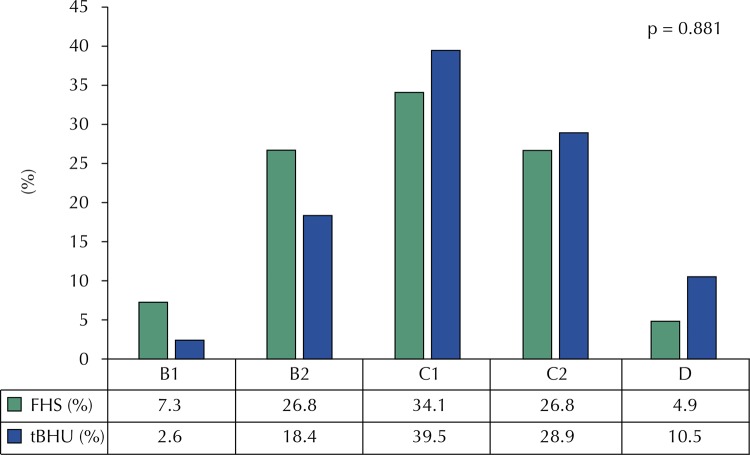
Socioeconomic status of the families of at-risk infants, according to the BHU management model they attend.

Figure 2 shows the socioeconomic status of families. Most of them belongs to the class C1, with no statistically significant difference between FHS and tBHU.

**Figure 3:**
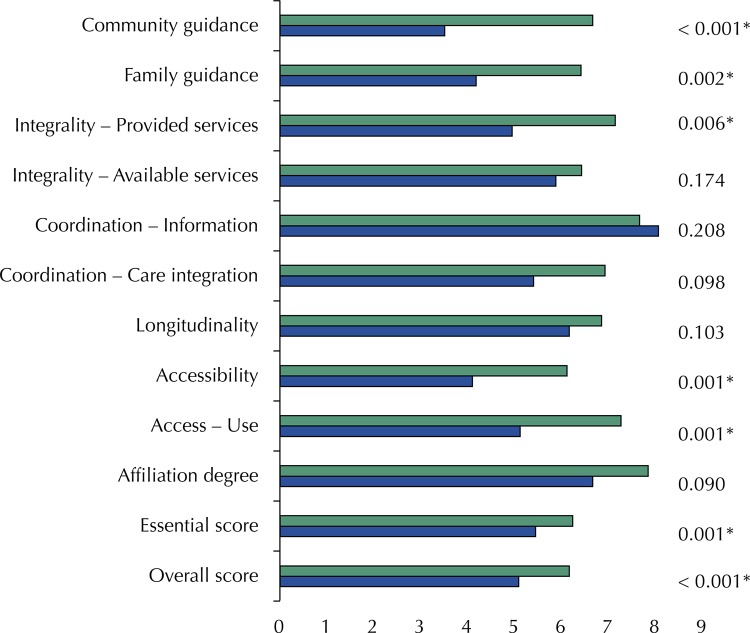
Average score values for each attribute, as well as overall score and essential score, according to the management model of basic health units. FHS: Family Health Strategy; tBHU: Traditional Basic Health Units

Regarding the overall and essential scores of PCATool – child version, the FHS were best assessed when compared with tBHU, with statistically significant difference for both scores. However, these scores are below 6.6 in both management models ( Figure 3 ).

When considering each attribute individually, FHS showed better performance for all attributes except for coordination of information systems, with a statistically significant difference for five of the ten items assessed ( Figure 3 ). It is noteworthy that seven attributes reached values ≥ 6.6 in the FHS and only two attributes reached 6.6 in tBHU.

Table 1 shows the score obtained on questions comprising each of the attributes, for both BHU models.

**Table 1:** Descriptive statistics and comparison between scores obtained in each question of PCATool – child version, according to the management model of the analyzed services.

A					B			
1^st^ Access contact – Use								

	Median and IQR (25–75%)			p		≥ 6.6 (%)		p
	
	tBHU	FHS				tBHU	FHS	
B1	10 (6.7–10.0)	10 (10.0–10.0)		0.075		76.9	88.1	0.243
B2	0 (0–5.0)	8.3 (0–10.0)		0.002*		25.6	64.3	0.001*
B3	10 (0–10.0)	10 (3.3–10.0)		0.711		56.4	69.0	0.259

Accessibility								
	Median and IQR (25–75%)			p		≥ 6.6 (%)		p
	
	tBHU	FHS				tBHU	FHS	

C1	3.3 (1.6–8.3)	10 (3.3–10.0)		0.002*		35.9	73.2	0.001*
C2	10 (0–10.0)	10 (3.3–10.0)		0.936		64.1	69.0	0.647
C3	6.7 (0–10.0)	10 (0–10.0)		0.187		51.3	65.9	0.256
C4	0 (0–6.7)	6.7 (0–10.0)		0.009*		28.2	57.1	0.013*
C5	10 (0–10.0)	10 (3.3–10.0)		0.455		56.4	61.0	0.821
C6	0 (0–10.0)	3.3 (0–3.3)		0.361		28.2	24.4	0.801

Longitudinality								

	Median and IQR (25–75%)			p		≥ 6.6 (%)		p
	
	tBHU	FHS				tBHU	FHS	

D1	10 (10.0–10.0)	10 (3.3–10.0)		0.002*		94.9	73.8	0.014*
D2	0 (0–3.3)	3.3 (0–6.7)		0.118		23.1	31.0	0.463
D3	10 (10.0–10.0)	10 (10.0–10.0)		0.950		87.2	90.5	0.732
D4	10 (10.0–10.0)	10 (10.0–10.0)		0.579		87.2	92.9	0.472
D5	10 (10.0–10.0)	10 (10.0–10.0)		0.365		82.1	90.5	0.339
D6	10 (10.0–10.0)	10 (10.0–10.0)		0.276		82.1	90.5	0.339
D7	3.3 (0–10.0)	10 (6.7–10.0)		0.003*		46.2	76.2	0.007*
D8	10 (10.0–10.0)	10 (3.3–10.0)		0.225		84.6	73.8	0.282
D9	10 (10.0–10.0)	10 (6.7–10.0)		0.633		84.6	81.0	0.772
D10	6.7 (0–10.0)	10 (0–10.0)		0.162		51.3	64.3	0.266
D11	0 (0–10.0)	3.3 (0–10.0)		0.078		28.2	47.6	0.109
D12	0 (0–6.7)	3.3 (0–10.0)		0.108		35.9	47.6	0.368
D13	0 (0–10.0)	1.6 (0–10.0)		0.515		35.9	40.5	0.819
D14	0 (0–10.0)	6.7 (0–10.0)		0.115		38.5	54.8	0.183

Coordination – Care integration								

	Median and IQR (25–75%)			p		≥ 6.6 (%)		p
	
	tBHU	FHS				tBHU	FHS	

E2	0 (0–10.0)	0 (0–10.0)		0.985		35.3	39.1	0.787
E3	10 (10.0–10.0)	10 (10.0–10.0)		0.980		85.3	82.6	1.000
E4	10 (0–10.0)	10 (10.0–10.0)		0.353		67.6	77.3	0.550
E5	10 (0–10.0)	10 (0–10.0)		0.316		55.9	72.7	0.263
E6	6.7 (0–10.0)	10 (10.0–10.0)		0.030*		52.9	81.8	0.045*

Coordination – Information systems								

	Median and IQR (25–75%)			p		≥ 6.6 (%)		p
	
	tBHU	FHS				tBHU	FHS	

F1	10 (10.0–10.0)	10 (10.0–10.0)		0.241		94.9	82.9	0.156
F2	10 (10.0–10.0)	10 (10.0–10.0)		0.341		86.5	97.5	0.100
F3	6.7 (3.3–10.0)	5 (3.3–6.7)		0.252		62.2	50.0	0.360

Integrality – Available services								

	Median and IQR (25–75%)			p		≥ 6.6 (%)		p
	
	tBHU	FHS				tBHU	FHS	

G1	10 (10.0–10.0)	10 (10.0–10.0)		0.505		97.4	100.0	0.481
G2	3.3 (0–10.0)	10 (3.3–10.0)		0.169		48.6	64.9	0.241
G3	10 (10.0–10.0)	10 (10.0–10.0)		0.530		86.1	87.8	1.000
G4	3.3 (0–3.3)	10 (1.7–10.0)		0.010*		20.0	57.1	0.003*
G5	3.3 (3.3–10.0)	3.3 (0–8.3)		0.520		42.4	45.7	0.812
G6	6.7 (3.3– 6.7)	3.3 (3.3–10.0)		0.955		52.9	42.9	0.473
G7	10 (3.3–10.0)	6.7 (3.3–10.0)		0.472		67.9	59.0	0.476
G8	6.7 (3.3–10.0)	10 (3.3–10.0)		0.090		52.9	60.0	0.330
G9	3.3 (0–3.3)	3.3 (1.7–6.7)		0.050*		14.3	35.1	0.057

Integrality – Provided services								

	Median and IQR (25–75%)			p		≥ 6.6 (%)		p
	
	tBHU	FHS				tBHU	FHS	

H1	10 (6.7–10.0)	10 (10.0–10.0)		0.241		87.2	90.5	0.732
H2	0 (0–10.0)	10 (0–10.0)		0.005*		35.9	68.3	0.007*
H3	10 (0–10.0)	10 (10.0–10.0)		0.109		64.1	76.2	0.330
H4	0 (0–10.0)	10 (0–10.0)		0.355		43.6	53.7	0.382
H5	3.3 (0–10.0)	10 (0–10.0)		0.029*		48.7	70.7	0.067

Family guidance								

	Median and IQR (25–75%)			p		≥ 6.6 (%)		p
	
	tBHU	FHS				tBHU	FHS	

I1	0 (0–10.0)	3.3 (0–10.0)		0.823		43.6	46.3	0.826
I2	10 (0–10.0)	10 (10.0–10.0)		0.299		69.2	80.5	0.305
I3	3.3 (0–6.7)	6.7 (3.3–10.0)		0.001*		43.6	68.3	0.042*

Community guidance								

	Median and IQR (25–75%)			p		≥ 6.6 (%)		p
	
	tBHU	FHS				tBHU	FHS	

J1	0 (0–10.0)	10 (10.0–10.0)		< 0.001*		43.6	87.2	0.001*
J2	3.3 (0–6.7)	10 (3.3–10.0)		0.003*		41.0	71.1	0.011*
J3	3.3 (0–10.0)	6.7 (3.3–10.0)		0.392		48.7	62.2	0.258
J4	0 (0–10.0)	3.3 (0–10.0)		0.228		41.0	45.9	0.817

* p ≤ 0.05 = statistically significant.

FHS: Family Health Strategy; tBHU: Traditional Basic Health Unit

A statistically significant difference was found in first contact access to the question about the search for the reference health service before going to another one, with better scores and a greater percentage of responses scored ≥ 6.6 for the FHS.

For the accessibility attribute, two questions showed statistically significant differences when comparing BHU, with better performance in FHS (same day service; waiting time). Higher percentages of responses scored ≥ 6.6 for FHS were observed.

As to longitudinality, two questions showed statistically significant difference: same health professional assistance, the performance was better in tBHU (score and percentages ≥ 6.6); however, as for the health professional knowledge, the performance was better in FHS (score and percentages).

Regarding coordination of care integration, the question of health professional interest in quality of care provided showed better performance in the FHS (score and answers percentages ≥ 6.6), with a statistically significant difference. To attribute coordination of information systems, no differences were found for any question.

For the attribute integrality – available services, both questions with statistically significant differences showed better results in the FHS (nutritional supplementation and assessment of visual problems). Moreover, for the attribute integrality – provided services, two questions showed significant differences, with better performance in the FHS (safekeeping of medicines and ways to keep the child safe).

Regarding family and community orientation, three questions were better scored in the FHS, with a statistically significant difference for the score and the percentage of responses scored ≥ 6.6 (if necessary, the health professional would meet with other family members; household visits; knowledge of common health problems in the neighborhood).

## DISCUSSION

To monitor growth and development in early childhood by PHC, an organized structure of HCN is necessary to enable assessment and classification of risks evenly across all services^2^ . The essential attributes of PHC, when recognized and practiced, directly reflect the effectiveness of health care and the provision of more effective and satisfactory service to population, with lower and fairer costs^7^ . The user perspective is one of the most reliable ways of measuring the quality of health services^12^ .

As to the socio-economic profile of the study population, the majority is in the C category^11^ , corroborating results of recent studies^12 , 13^ .

In the comparison of management models of PHC services, FHS was best assessed in all attributes except for coordination of information systems. These data confirm previous study, which indicated higher quality in care and provision of services and information of FHS on health care for the child^3^ . However, overall and essential scores of both models showed values below those considered appropriate in this study.

In coordination of information systems, answers were similar for both models, indicating that, in general, at the child’s appointment, the medical chart was present, and the guardian could look into it. A positive assessment of this attribute shows health records are being used as communication tools in services^14^ . Different results were found in a previous study, in which FHS was better assessed, indicating greater efficiency in records organization^3^ .

As to first contact access – use, the FHS was better assessed, especially regarding the demand of the service in a new health problem (before going to other treatment site). The operation of tBHU, with appointment scheduling only with the pediatrician, seems to contribute to the search for another service. There is also a culture of looking for specialized services in an emergency, trusting them to be more effective^16^ . The gateway to a health service should be easily accessible to any healthcare level^15^ . From the collected results, PHC still has limitations to satisfy the population’s needs, being smaller in FHS and larger in tBHU. However, this attribute was one of the best assessed in FHS, showing that management model is recognized as a health service reference, both for routine appointment and emergencies.

The attribute accessibility was the worst rated in FHS and the second worst in tBHU, showing the difficulty of getting and appointment for the same day and/or guidance by phone and/or high waiting time. The lack of efficiency in this attribute can hinder the resolution and performance of health services. An affordable service is the one easy to contact, available to users without geographical, administrative, financial and sociocultural barriers^17^ . The low scores obtained can be attributed to BHU working hours, which close early on weekdays and are close on weekends^16 , 17^ . For both questions, the performance of FHS was better (same day service and waiting time); the major obstacle in tBHU is the child’s appointment only by a pediatrician, who often is not in service or it has a waiting queue.

For longitudinality, FHS was better assessed, despite being one of the best attributes assessed in tBHU. Positive results for regular care by the same professional, in both models (but better assessed in tBHU) suggest the establishment of a strong bond of users with the units, which recognize BHU as a regular source of care and reference to health needs. This translates into a relationship of responsibility and trust among the health team, enabling more accurate diagnoses and more effective treatments^15^ .

Interaction between health professionals, caregiver/guardian and child’s medical history is an essential aspect of quality care. The answers on the knowledge of professionals about the children’s medical history and behavior were positive, suggesting that, in both models, this aspect is appreciated for care planning. However, data relating to child’s recognition as a person and not just as a health problem show that the care provided is not consistent with the PHC principles. We also observed a significant discrepancy between services, as FHS was the better assessed. It is noteworthy that FHS is following the guidelines proposed to provide comprehensive care to the users in their social and familiar context^18^ . In contrast, data for tBHU reflect the hegemony of the biomedical and curative care model.

Negative results were observed in FHS and tBHU on professionals’ knowledge about family, indicating that although the longitudinality assessment have been positive in FHS, health practices aimed at the family knowledge context are still fragile, reinforcing the biomedical care model^19^ . Therefore, it is necessary to develop the relationship between professionals and users, increasing the satisfaction and resolution of population’s needs^20^ , changing the work process of tBHU, and seeking for care quality and effective implementation of the guidelines proposed in PHC.

To the attribute coordination – care integration, FHS was better assessed, especially regarding the health professional interest about the care provided to the infant in specialty outpatient clinic. This suggests that FHS provides more care integration and trust between users and health professionals, corroborating previous study results^3^ . To a satisfactory coordination, PHC and secondary care/specialist need to maintain adequate communication, as well as an efficient reference/counter-reference system^21 , 22^ . In the tBHU case, it is still necessary to improve the integration and coordination between PHC and secondary level of region to speed up the reference/counter-reference flow.

The attribute integrality of available services showed no difference between the services examined, which were below the cutoff point. However, FHS was better rated about services provision – nutritional supplementation and identification of visual problems. A previous study identified best score for FHS regarding the integrality in child’s health care^3^ . This result shows that FHS has become a management model that seeks compliance with guidelines related to this attribute of PHC^23^ .

Prevention of childhood obesity, especially by actions in PHC, is one of the World Health Organization priorities^24^ . The recommended actions aim at breastfeeding and healthy eating in order to develop healthy satiety in children and prevent the occurrence of changes in the child’s growth and development^23 , 24^ .

We observed that even with scores for nutritional support below adequate, in FHS users knew more about existing programs, which increases the nutritional health care to at-risk infants. In contrast, previous studies have identified scores below appropriate for this attribute, referring specifically to the provision of nutritional supplementation programs for both FHS and tBHU^3 , 23^ . We must emphasize that, without proper support of local health professionals, families in higher social and environmental vulnerability will have more difficulties to join programs related to nutritional supplementation, creating health risks to infants^24^ .

PHC also includes actions to prevent visual changes and accuracy assessment, in partnership with schools^23^ . A higher knowledge of FHS users about these services and greater availability of these, compared with tBHU was verified.

The inadequate overall performance of the integrality of services may indicate a real difficulty to offer a full range of actions and resources on the health needs of users^21 - 23^ . Thus, it is necessary to invest continuously in physical, material and humans resources to achieve integration within the health services^21 , 22^ . Constantly, this question comes as a problem of difficult resolution within the public service, as many municipalities show instability in the provision of their own resources and state and federal funding, compromising the health system autonomy and development of actions in PHC^23^ .

Concerning to services provided, the score was better for the FHS (≥ 6.6), indicating that services provided to children are deficient in the tBHU model, confirming results from previous study^25^ .

Thus, some important topics to child’s health are not being consistently addressed during appointments in tBHU. Among them, questions about home safety and ways to deal with child behavior problems, which received very low scores. A previous study found that a considerable part of the analyzed sample does not receive guidance on these issues, showing services fragility, since these are soft technologies with low cost^4^ . Other studies have found similar results, showing that childcare is fragmented, leaving aside the broader factors that affect the child’s health^23 , 26^ .

As to the specific issue of child growth and development, we observed suitable scores for both models. This question is important for monitoring risk infants, since there is an increased chance of perinatal and childhood diseases in this population. The at-risk newborn must be accompanied in PHC until the second year of life, aiming to health promotion, protection and early detection of changes that may affect the child’s quality of life^27 , 28^ .

One hypothesis for the high score detected is the monitoring of child’s growth in PHC, usually addressed in a consistent way^27^ , since it is linked to commonly emphasized biological factors^23^ . As for development, previous study found that over 56% of records gathered in FHS and tBHU showed no information relating to development milestones^27^ , suggesting that monitoring might not be effective. This potential weakness may be masked by the simultaneous approach of these two aspects in the same question^29^ .

The results of this study related to integrality (available services and provided services) corroborate previous studies that showed that, even in FHS, this attribute still falls short of expectations^23 , 30^ . This population is still in a situation of vulnerability, which can cause harm to the child’s growth and development, requiring greater efforts to be undertaken by professionals and managers within the organization and coordination of PHC services^23^ .

As to family and community orientation, only FHS reached appropriate average. In relation to childcare, scores were < 6.6, indicating that the approach between health services and families is insufficient for both management models^3^ . This same trend was observed in the knowledge of community health problems, especially in tBHU, suggesting childcare may be detached from the community reality^3^ . These findings may also result from the lack of care model assimilation centered on family and community^12^ . These attributes are expected to be enhanced in FHS, which may explain the better assessment of this model.

A systematic review examined the assessment of PHC attributes made by BHU users via PCATool. In general, the attribute first contact access showed low scores. Longitudinality was well assessed in most studies, as well as integrality, which is superior in FHS in some of them, as well as coordination. For the attributes family and community orientation, the performance was below the expected in almost all studies; however, they showed higher scores in FHS^21^ .

This study corroborates the results of the systematic review^21^ , noting that FHS shows better overall performance. As for the attributes, tBHU showed low scores in all of them, except coordination of information systems. FHS had low scores in accessibility, integrality and family orientation, differing from some results of the review.

We observed lower overall and essential scores in both management models, which shows that guardians of at-risk infants assessed some attributes as unsatisfactory, emphasizing accessibility, integrality and family orientation. These attributes are considered essential to PHC, and their underperformance may bring negative consequences for the quality and comprehensive care of at-risk infants, since it is up to the PHC service to create mechanisms to meet the person’s needs^19^ . All these actions must put family as the subject of care to integrate the at-risk infant healthcare in a familiar and community approach^15 , 21^ .

It is noteworthy that the best performance of FHS, although there are difficulties in everyday BHU, reflects the investment made in this management model in recent years to restructure PHC.
