# Supplementary material for: Evaluation of at-risk infant care: comparison between models of primary health care
Source: Rev Saude Publica. 2019 Nov 12;53:98. doi: 10.11606/s1518-8787.2019053001063 (PMC6855796; doi:10.11606/s1518-8787.2019053001063)
Supplement: Translation

# Translation: Avaliação do cuidado ao bebê de risco: comparação de modelos de atenção primária à saúde

**Authors:** Alessandra Giannella Samelli, Gislene Andrade Tomazelli, Maria Helena Morgani de Almeida, Fátima Corrêa Oliver, Silmara Rondon-Melo, Daniela Regina Molini-Avejonas

**Affiliations:** I Universidade de São Paulo. Faculdade de Medicina. Curso de Fonoaudiologia do Departamento de Fisioterapia, Fonoaudiologia e Terapia Ocupacional. São Paulo, SP, Brasil; II Universidade de São Paulo. Faculdade de Medicina. Curso de Terapia Ocupacional do Departamento de Fisioterapia, Fonoaudiologia e Terapia Ocupacional. São Paulo, SP, Brasil

**Keywords:** Recém-Nascido, Grupos de Risco, Atenção Primária à Saúde, Qualidade, Acesso e Avaliação da Assistência à Saúde, Estratégia Saúde da Família, Sistema Único de Saúde

## Abstract

**OBJETIVOS:**

Analisar a rede de cuidados ao bebê de risco na região Oeste do município de São Paulo, tendo a atenção primária à saúde como ordenadora, e comparar a presença e extensão dos atributos da atenção primária à saúde nos serviços prestados, de acordo com o modelo de gestão dos serviços (Estratégia Saúde da Família e unidade básica de saúde tradicional).

**MÉTODOS:**

Foi feito levantamento de todos os bebês de risco nascidos na região entre 2013 e 2014. Em seguida, fez-se busca ativa dessas crianças para posterior aplicação do questionário PCATool versão criança. Foram localizadas no território 233 crianças; 113 responsáveis por crianças concordaram em participar do estudo, e 81 compuseram a amostra final.

**RESULTADOS:**

Quanto aos resultados do PCATool para escores geral e essencial, as unidades com Estratégia Saúde da Família foram mais bem avaliadas pelos usuários quando comparadas com as unidades básicas de saúde tradicional, com diferença estatisticamente significante. Entretanto, estes escores apresentaram-se baixos para ambos os modelos de gestão. Quanto aos atributos, a Estratégia Saúde da Família apresentou desempenho melhor quando comparada à unidade básica de saúde tradicional para a maioria deles, com exceção de coordenação dos sistemas de informação. Dos dez atributos avaliados, sete atingiram valores ≥ 6,6 para a Estratégia Saúde da Família e dois, para unidade básica de saúde tradicional.

**CONCLUSÕES:**

Independentemente do modelo de gestão, verificaram-se baixos escores geral e essencial, indicando que os responsáveis pelos bebês de risco avaliaram como insatisfatórios alguns atributos, com destaque para acessibilidade, integralidade e orientação familiar. Esse desempenho pode trazer consequências negativas para a qualidade e integralidade do cuidado ao bebê de risco.

## INTRODUÇÃO

As Redes de Atenção à Saúde (RAS) são conjuntos de serviços de saúde vinculados entre si, com missão e objetivos comuns. Ofertam atenção contínua e integral à determinada população, coordenada pela atenção primária à saúde (APS), compondo sistemas de saúde funcionais^1^ . Nessa perspectiva, o Ministério da Saúde (MS) definiu quatro redes temáticas prioritárias: Cegonha, Atenção às urgências e emergências, Atenção psicossocial e Cuidado à pessoa com deficiência^2^ . A Rede Cegonha foi implementada para assegurar a assistência humanizada a gestantes e o acompanhamento integral da criança em seus dois primeiros anos de vida. Deve garantir a vinculação do binômio mãe-bebê, promover a atenção integral à saúde com qualidade e resolutividade e realizar a busca ativa de crianças em situação de vulnerabilidade.

A Estratégia Saúde da Família (ESF) foi criada na perspectiva de mudar o modo tradicional de prestação da assistência em saúde, com o objetivo de fomentar um modelo com a APS na coordenação do cuidado, respeitando os princípios de integralidade nas relações familiares e comunitárias, de universalização e de equidade^3 , 4^ . Embora estudos prévios indiquem maior satisfação dos usuários com a ESF, ainda é necessário ampliar as avaliações sobre a gestão e a oferta de serviços e do cuidado, utilizando-se questionários padronizados e validados^3^ . O MS tem desenvolvido estratégias para avaliação e monitoramento da APS, considerando também a satisfação ou percepção de profissionais e usuários. Um processo de avaliação desse tipo possibilita direcionamento no momento de tomada das decisões nos serviços, visando a transformar a APS em porta de entrada, com a confiabilidade e resolutividade esperadas para uma rede de atenção de qualidade^5^ .

O instrumento *Primary Care Assessment Tool* (PCATool) foi criado com base em um modelo de avaliação da qualidade de serviços de saúde, mensurando aspectos de estrutura, processo e resultados^6^ . O PCATool tem como objetivo avaliar atributos essenciais e derivados da APS em serviços prestados a adultos e crianças, relacionando as experiências do usuário com os profissionais e com o serviço de saúde, medindo sua satisfação. O instrumento foi traduzido, adaptado e validado para o português brasileiro e é utilizado para avaliar a APS^7 , 8^ .

O objetivo deste estudo foi analisar a rede de cuidados ao bebê de risco na região Oeste do município de São Paulo, tendo a APS como ordenadora do cuidado. Como objetivos específicos, pretendeu-se comparar a presença e extensão dos atributos da APS nos serviços prestados aos bebês de risco de diferentes modelos de gestão (ESF e tradicional).

Foram definidas como hipóteses deste estudo:

1. As UBS com ESF serão mais bem avaliadas quando comparadas com as UBS de modelo tradicional (UBSt), e os escores ficarão acima de 6,6.
2. Ao considerar cada um dos atributos individualmente, a ESF apresentará melhor desempenho, principalmente nos atributos: acesso de primeiro contato, acessibilidade, longitudinalidade, coordenação da integração de cuidados, e orientação familiar e comunitária.

## MÉTODOS

Estudo transversal e descritivo, aprovado pelos Comitês de Ética em Pesquisa da Faculdade de Medicina da Universidade de São Paulo (FMUSP) (189/14) e da Prefeitura de São Paulo (32273014.8.3001.0086). Os responsáveis pelos bebês de risco assinaram o Termo de Consentimento Livre e Esclarecido.

Foi realizada busca de todos os recém-nascidos classificados como de risco na região Oeste da cidade de São Paulo, Brasil (coordenada pela Supervisão Técnica de Saúde do Butantã – STS/BT), entre 1 de agosto de 2013 a 28 de fevereiro de 2014, por meio das declarações dos nascidos vivos (DNV).

Foram utilizados os critérios de classificação de bebê de risco determinados pelo Decreto Nº 43.407^9^ , visto que os sujeitos pertenciam à STS/BT, que possuía uma demanda específica referente ao diagnóstico territorial de caracterização desses bebês. Os critérios de risco adotados pela STS/BT são: peso ao nascer ≤ 2.500 g, Apgar no 1º minuto de vida ≤ 5 e idade materna ≤ 16 anos de idade. Para ser incluído no estudo, o bebê deveria apresentar ao menos um desses critérios de risco. Assim, houve algumas diferenças com relação aos riscos descritos na cartilha do MS^10^ .

A STS/BT tem população estimada em 442.198 habitantes, sendo 31.924 crianças entre 0 e 4 anos. Conta com 14 unidades básicas de saúde (UBS), sendo sete ESF (com o apoio de dois NASF) e sete tradicionais (UBSt), um ambulatório de especialidades e dois hospitais-maternidades como serviços de referência para os bebês de risco nascidos/residentes no território. A população-alvo compreendeu os responsáveis pelos bebês de risco dessa região, cadastrados em uma das UBS e selecionados conforme a Figura 1 .

**Figura 1:** Fluxo de seleção dos participantes do estudo.

Apenas os dados referentes à avaliação da APS foram incluídos; assim, foram excluídos 32 sujeitos que optaram por responder ao PCATool referindo-se ao serviço secundário. Portanto, a amostra final foi composta por 81 participantes.

Primeiramente, aplicou-se o questionário socioeconômico da Associação Brasileira de Empresas de Pesquisa^11^ . Em seguida, foi aplicado o PCATool – versão criança^6^ , que avalia os seguintes atributos essenciais da APS: acesso de primeiro contato (acessibilidade e uso de serviços); longitudinalidade (fonte regular de cuidados e uso ao longo do tempo); integralidade (serviços disponíveis e voltados à atenção integral, incluindo encaminhamentos a outros serviços) e coordenação de cuidados (continuidade e reconhecimento de problemas tratados em outros serviços, bem como integração desses cuidados). Os atributos derivados avaliados qualificam as ações dos serviços: orientação familiar e comunitária^6^ .

O pressuposto da distribuição normal de cada variável foi avaliado pelo teste Shapiro-Wilk. As análises descritivas das variáveis numéricas foram apresentadas por meio de médias, medianas e intervalos interquartis (IQ); as variáveis categóricas, por meio de porcentagens.

Como testes de hipótese, foram utilizados: Mann-Whitney (variáveis numéricas), qui-quadrado ou exato de Fisher (variáveis categóricas), assumindo um nível de significância de p ≤ 0,05. As análises foram realizadas no software SPSS 21 (Windows).

## RESULTADOS

Dos 81 bebês participantes, 44 eram do sexo masculino (54,32%) e 37, do feminino (45,67%). Quanto ao modelo de gestão, 42 bebês (51,85%) estavam vinculados à ESF e 39 (48,15%), à UBSt.

A Figura 2 apresenta os níveis socioeconômicos das famílias. Nota-se que a maioria pertence à classe C1, sem diferença estatisticamente significante entre ESF e UBSt.

**Figura 2:** Nível socioeconômico das famílias dos bebês de risco, de acordo com o modelo de gestão das UBS das quais são usuárias.

Em relação aos escores geral e essencial do PCATool – versão criança, a ESF foi mais bem avaliada quando comparada com a UBSt, com diferença estatisticamente significante para ambos os escores. Entretanto, observa-se que esses escores estão abaixo de 6,6 em ambos os modelos de gestão ( Figura 3 ).

**Figura 3:** Valores médios dos escores para cada um dos atributos, bem como escore geral e escore essencial, segundo modelo de gestão das unidades básicas de saúde. ESF: Estratégia Saúde da Família; UBSt: unidade básica de saúde de modelo tradicional.

Ao considerar cada um dos atributos individualmente, a ESF apresentou melhor desempenho para todos os atributos, exceto para coordenação dos sistemas de informação, com diferença estatisticamente significante para cinco dos dez itens avaliados ( Figura 3 ). Destaca-se que sete atributos alcançaram valores ≥ 6,6 na ESF e apenas dois atributos atingiram 6,6 na UBSt.

A Tabela 1 mostra a pontuação obtida nas questões que compuseram cada um dos atributos, para os dois modelos de UBS.

**Tabela 1:** Estatística descritiva e comparação entre os escores obtidos em cada questão do PCATool – versão criança, segundo o modelo de gestão dos serviços analisados.

A					B			
Acesso 1º Contato – Utilização								

	Mediana e IQ (25–75%)			p		≥ 6,6 (%)		p
	
	UBSt	ESF				UBSt	ESF	

B1	10 (6,7–10,0)	10 (10,0–10,0)		0,075		76,9	88,1	0,243
B2	0 (0–5,0)	8,3 (0–10,0)		0,002*		25,6	64,3	0,001*
B3	10 (0–10,0)	10 (3,3–10,0)		0,711		56,4	69,0	0,259

Acessibilidade								

	Mediana e IQ (25–75%)			p		≥ 6,6 (%)		p
	
	UBSt	ESF				UBSt	ESF	

C1	3,3 (1,6–8,3)	10 (3,3–10,0)		0,002*		35,9	73,2	0,001*
C2	10 (0–10,0)	10 (3,3–10,0)		0,936		64,1	69,0	0,647
C3	6,7 (0–10,0)	10 (0–10,0)		0,187		51,3	65,9	0,256
C4	0 (0–6,7)	6,7 (0–10,0)		0,009*		28,2	57,1	0,013*
C5	10 (0–10,0)	10 (3,3–10,0)		0,455		56,4	61,0	0,821
C6	0 (0–10,0)	3,3 (0–3,3)		0,361		28,2	24,4	0,801

Longitudinalidade								

	Mediana e IQ (25–75%)			p		≥ 6,6 (%)		p
	
	UBSt	ESF				UBSt	ESF	

D1	10 (10,0–10,0)	10 (3,3–10,0)		0,002*		94,9	73,8	0,014*
D2	0 (0–3,3)	3,3 (0–6,7)		0,118		23,1	31,0	0,463
D3	10 (10,0–10,0)	10 (10,0–10,0)		0,950		87,2	90,5	0,732
D4	10 (10,0–10,0)	10 (10,0–10,0)		0,579		87,2	92,9	0,472
D5	10 (10,0–10,0)	10 (10,0–10,0)		0,365		82,1	90,5	0,339
D6	10 (10,0–10,0)	10 (10,0–10,0)		0,276		82,1	90,5	0,339
D7	3,3 (0–10,0)	10 (6,7–10,0)		0,003*		46,2	76,2	0,007*
D8	10 (10,0–10,0)	10 (3,3–10,0)		0,225		84,6	73,8	0,282
D9	10 (10,0–10,0)	10 (6,7–10,0)		0,633		84,6	81,0	0,772
D10	6,7 (0–10,0)	10 (0–10,0)		0,162		51,3	64,3	0,266
D11	0 (0–10,0)	3,3 (0–10,0)		0,078		28,2	47,6	0,109
D12	0 (0–6,7)	3,3 (0–10,0)		0,108		35,9	47,6	0,368
D13	0 (0–10,0)	1,6 (0–10,0)		0,515		35,9	40,5	0,819
D14	0 (0–10,0)	6,7 (0–10,0)		0,115		38,5	54,8	0,183

Coordenação – Integração de cuidados								

	Mediana e IQ (25–75%)			p		≥ 6,6 (%)		p
	
	UBSt	ESF				UBSt	ESF	

E2	0 (0–10,0)	0 (0–10,0)		0,985		35,3	39,1	0,787
E3	10 (10,0–10,0)	10 (10,0–10,0)		0,980		85,3	82,6	1,000
E4	10 (0–10,0)	10 (10,0–10,0)		0,353		67,6	77,3	0,550
E5	10 (0–10,0)	10 (0–10,0)		0,316		55,9	72,7	0,263
E6	6,7 (0–10,0)	10 (10,0–10,0)		0,030*		52,9	81,8	0,045*

Coordenação – Sistemas de informação								

	Mediana e IQ (25–75%)			p		≥ 6,6 (%)		p
	
	UBSt	ESF				UBSt	ESF	

F1	10 (10,0–10,0)	10 (10,0–10,0)		0,241		94,9	82,9	0,156
F2	10 (10,0–10,0)	10 (10,0–10,0)		0,341		86,5	97,5	0,100
F3	6,7 (3,3–10,0)	5 (3,3–6,7)		0,252		62,2	50,0	0,360

Integralidade – Serviços disponíveis								

	Mediana e IQ (25–75%)			p		≥ 6,6 (%)		p
	
	UBSt	ESF				UBSt	ESF	

G1	10 (10,0–10,0)	10 (10,0–10,0)		0,505		97,4	100,0	0,481
G2	3,3 (0–10,0)	10 (3,3–10,0)		0,169		48,6	64,9	0,241
G3	10 (10,0–10,0)	10 (10,0–10,0)		0,530		86,1	87,8	1,000
G4	3,3 (0–3,3)	10 (1,7–10,0)		0,010*		20,0	57,1	0,003*
G5	3,3 (3,3–10,0)	3,3 (0–8,3)		0,520		42,4	45,7	0,812
G6	6,7 (3,3– 6,7)	3,3 (3,3–10,0)		0,955		52,9	42,9	0,473
G7	10 (3,3–10,0)	6,7 (3,3–10,0)		0,472		67,9	59,0	0,476
G8	6,7 (3,3–10,0)	10 (3,3–10,0)		0,090		52,9	60,0	0,330
G9	3,3 (0–3,3)	3,3 (1,7–6,7)		0,050*		14,3	35,1	0,057

Integralidade – Serviços prestados								

	Mediana e IQ (25–75%)			p		≥ 6,6 (%)		p
	
	UBSt	ESF				UBSt	ESF	

H1	10 (6,7–10,0)	10 (10,0–10,0)		0,241		87,2	90,5	0,732
H2	0 (0–10,0)	10 (0–10,0)		0,005*		35,9	68,3	0,007*
H3	10 (0–10,0)	10 (10,0–10,0)		0,109		64,1	76,2	0,330
H4	0 (0–10,0)	10 (0–10,0)		0,355		43,6	53,7	0,382
H5	3,3 (0–10,0)	10 (0–10,0)		0,029*		48,7	70,7	0,067

Orientação familiar								

	Mediana e IQ (25–75%)			p		≥ 6,6 (%)		p
	
	UBSt	ESF				UBSt	ESF	

I1	0 (0–10,0)	3,3 (0–10,0)		0,823		43,6	46,3	0,826
I2	10 (0–10,0)	10 (10,0–10,0)		0,299		69,2	80,5	0,305
I3	3,3 (0–6,7)	6,7 (3,3–10,0)		0,001*		43,6	68,3	0,042*

Orientação comunitária								

	Mediana e IQ (25–75%)			p		≥ 6,6 (%)		p
	
	UBSt	ESF				UBSt	ESF	

J1	0 (0–10,0)	10 (10,0–10,0)		< 0,001*		43,6	87,2	0,001*
J2	3,3 (0–6,7)	10 (3,3–10,0)		0,003*		41,0	71,1	0,011*
J3	3,3 (0–10,0)	6,7 (3,3–10,0)		0,392		48,7	62,2	0,258
J4	0 (0–10,0)	3,3 (0–10,0)		0,228		41,0	45,9	0,817

* p ≤ 0,05 = estatisticamente significante.

ESF: Estratégia Saúde da Família; UBSt: unidade básica de saúde de modelo tradicional.

Verificou-se diferença estatisticamente significante no acesso de primeiro contato, para a questão sobre a procura pelo serviço de saúde de referência antes de ir a outro serviço, com melhor escore e maior porcentagem de respostas pontuadas ≥ 6,6 para a ESF.

Para o atributo acessibilidade, duas perguntas mostraram diferenças estatisticamente significantes na comparação entre UBS, com desempenho melhor na ESF (atendimento no mesmo dia; tempo de espera). Verificaram-se maiores porcentagens de respostas pontuadas ≥ 6,6 para ESF.

Quanto à longitudinalidade, também foram duas as questões com diferença estatisticamente significante: para atendimento pelo mesmo profissional de saúde, o desempenho foi melhor na UBSt (pontuação e porcentagens ≥ 6,6); já para conhecimento como pessoa por parte do profissional de saúde, o desempenho foi melhor na ESF (pontuação e porcentagens).

Em relação à coordenação da integração de cuidados, a questão sobre o interesse do profissional de saúde na qualidade do cuidado prestado no serviço especializado mostrou melhor desempenho na ESF (pontuação e porcentagens de respostas ≥ 6,6), com diferença estatisticamente significante. Para o atributo coordenação dos sistemas de informação, não houve diferença para nenhuma questão.

Para o atributo integralidade – serviços disponíveis, ambas as questões com diferenças estatisticamente significantes mostraram resultados melhores na ESF (suplementação nutricional e avaliação de problemas visuais). Também para o atributo integralidade – serviços prestados, duas questões mostraram diferenças significantes, com desempenho melhor na ESF (guarda de medicamentos e maneiras para manter a criança segura).

Quanto à orientação familiar e comunitária, três questões foram mais bem pontuadas na ESF, com diferença estatisticamente significante para o escore e para a porcentagem de respostas pontuadas ≥ 6,6 (se necessário, o profissional de saúde se reuniria com outros membros da família; visitas domiciliares; conhecimento sobre problemas de saúde na vizinhança).

## DISCUSSÃO

A fim de garantir o acompanhamento do crescimento e desenvolvimento na primeira infância pela APS, é necessária uma estruturação organizada da RAS, que permita a avaliação e a classificação dos riscos de forma homogênea em todos os serviços que a compõem^2^ . Os atributos essenciais da APS, quando reconhecidos e praticados, refletem diretamente na efetividade da atenção em saúde, sendo a prestação de serviço mais efetiva e satisfatória para a população, com custos menores e mais equitativos^7^ . A perspectiva do usuário é uma das maneiras mais fidedignas de mensurar a qualidade dos serviços de saúde^12^ .

Quanto ao perfil socioeconômico da população estudada, verificou-se que a maioria se encontra na categoria C^11^ , corroborando resultados de estudos recentes^12 , 13^ .

Na comparação entre os modelos de gestão dos serviços de APS, a ESF foi mais bem avaliada em todos os atributos, exceto para coordenação dos sistemas de informação. Esses dados corroboram estudo prévio, que indicou maior qualidade no cuidado e na oferta de serviços e informações da ESF na atenção à saúde da criança^3^ . Entretanto, os escores geral e essencial de ambos os modelos apresentaram valores abaixo do considerado adequado no presente estudo.

Na coordenação dos sistemas de informação, as respostas foram semelhantes para ambos os modelos, indicando que, em geral, no momento da consulta da criança, o prontuário esteve presente e o responsável poderia consultá-lo. Uma avaliação positiva desse atributo mostra que os registros de saúde estão sendo utilizados como instrumentos de comunicação nos serviços^14^ . Resultado diferente foi encontrado em estudo prévio, em que a ESF foi mais bem avaliada, indicando maior eficácia na organização dos registros^3^ .

Quanto ao acesso de primeiro contato – utilização, a ESF foi mais bem avaliada, principalmente quanto à procura do serviço em caso de novo problema de saúde (antes de ir a outro equipamento de saúde). A dinâmica do funcionamento da UBSt, com agendamento de consultas somente com o pediatra, parece contribuir para a procura por outro serviço. Há também uma cultura de procurar serviços especializados em caso de emergência, acreditando-se serem mais resolutivos^16^ . A porta de entrada para um serviço de saúde deve ser de fácil acesso para qualquer nível de atenção^15^ . Pelos resultados obtidos, a APS ainda apresenta limitações em atender as necessidades da população, sendo menores na ESF e maiores na UBSt. Contudo, ressalta-se que este atributo foi um dos mais bem avaliados na ESF, mostrando se tratar de um modelo de gestão reconhecido como serviço de saúde de referência, tanto em consultas de rotina como em emergências.

O atributo acessibilidade foi o pior avaliado na ESF e o segundo pior na UBSt, mostrando que houve dificuldade na obtenção de consulta para o mesmo dia e/ou orientações por telefone e/ou o tempo de espera foi elevado. A falta de eficiência nesse atributo pode dificultar a resolutividade e o desempenho dos serviços de saúde. Um serviço acessível é aquele de fácil contato, disponível aos usuários, sem barreiras geográficas, administrativas, financeiras e socioculturais^17^ . Os baixos escores obtidos podem ser atribuídos aos horários de funcionamento das UBS, que fecham cedo nos dias úteis e não abrem aos fins de semana^16 , 17^ . Para duas perguntas, o desempenho foi melhor para a ESF (atendimento no mesmo dia e tempo de espera); o grande obstáculo nas UBSt é a realização do atendimento da criança somente pelo pediatra, que, muitas vezes, não se encontra no serviço ou apresenta fila de espera.

Para longitudinalidade, a ESF foi mais bem avaliada, mesmo sendo um dos atributos mais bem avaliados nas UBSt. Os resultados positivos para regularidade de atendimento pelo mesmo profissional, em ambos os modelos (mas mais bem avaliado na UBSt), sugerem o estabelecimento de um forte vínculo dos usuários com as unidades, os quais reconhecem as UBS como uma fonte regular de cuidados e referência para atenção às necessidades de saúde. Isso se traduz em uma relação de responsabilidade e confiança na equipe de saúde, possibilitando diagnósticos mais precisos e tratamentos mais eficazes, com maior resolutividade^15^ .

A interação entre profissional de saúde, cuidador/responsável e história da criança é aspecto essencial para um atendimento de qualidade. As respostas sobre o conhecimento dos profissionais a respeito da história clínica das crianças e das condutas foram positivas, sugerindo que, em ambos os modelos, há a valorização desse aspecto para o planejamento do cuidado. No entanto, os dados referentes ao reconhecimento da criança como pessoa e não apenas como um problema de saúde mostram que a atenção não ocorre de forma coerente com os princípios da APS; observa-se, ainda, uma discrepância significativa entre os serviços, sendo a ESF mais bem avaliada. É notável que a ESF está seguindo as diretrizes propostas para a atenção integral ao usuário em seu contexto social e familiar^18^ . Em contrapartida, os dados para a UBSt refletem a hegemonia do modelo de atenção biomédico e curativo.

Resultados negativos foram observados na ESF e na UBSt sobre o conhecimento dos profissionais a respeito da família, indicando que, apesar de a avaliação da longitudinalidade ter sido positiva na ESF, as práticas de saúde voltadas ao conhecimento do contexto familiar ainda são frágeis, reforçando o modelo assistencial biomédico^19^ . Diante disso, é preciso estreitar a relação entre profissional e usuário, contribuindo para maior satisfação e resolutividade das necessidades da população^20^ , bem como fazer mudanças profundas no processo de trabalho das UBSt, buscando melhoria da qualidade da atenção e efetiva implementação das diretrizes propostas na APS.

Para o atributo coordenação da integração de cuidados, a ESF foi mais bem avaliada, sobretudo quanto ao interesse do profissional de saúde em saber sobre o cuidado prestado ao bebê no serviço de especialidades em que foi atendido. Isso sugere que na ESF há maior atenção à integração do cuidado e maior confiança entre usuários e profissionais de saúde, corroborando os resultados de estudo anterior^3^ . Para que ocorra uma coordenação satisfatória, é necessário que APS e atenção secundária/especializada mantenham comunicação adequada, bem como um sistema de referência/contrarreferência eficiente^21 , 22^ . No caso das UBSt, ainda é necessário melhorar a integração e a articulação entre APS e nível secundário da região para agilizar os fluxos de referência/contrarreferência.

O atributo integralidade dos serviços disponíveis não mostrou diferença entre os serviços analisados, os quais ficaram abaixo do ponto de corte. Contudo, a ESF foi mais bem avaliada quanto à oferta de serviços – suplementação nutricional e identificação de problemas visuais. Estudo prévio identificou melhor pontuação para a ESF quanto à integralidade no cuidado à saúde da criança^3^ . Esse resultado mostra que a ESF tem se tornado um modelo de gestão em que se busca o cumprimento das diretrizes relacionadas a esse atributo da APS^23^ .

A prevenção da obesidade na infância, sobretudo por meio de ações na APS, é uma das prioridades da Organização Mundial da Saúde^24^ . São preconizadas ações visando ao aleitamento materno e à alimentação saudável, a fim de desenvolver a saciedade saudável na criança e prevenir a ocorrência de alterações no crescimento e desenvolvimento infantil^23 , 24^ .

Observa-se que, mesmo com escore para suporte nutricional abaixo do adequado, na ESF houve maior conhecimento por parte dos usuários sobre programas existentes, o que faz aumentar as possibilidades de cuidado à saúde nutricional dos bebês de risco. Em contrapartida, estudos prévios identificaram escores abaixo do adequado para este atributo, referindo-se especificamente à oferta de programas de suplementação nutricional, tanto para ESF como para UBSt^3 , 23^ . É importante enfatizar que, sem o devido suporte dos profissionais de saúde locais, famílias em maior vulnerabilidade social e ambiental terão mais dificuldades para aderir a programas relacionados à suplementação nutricional, gerando riscos à saúde da criança^24^ .

A APS também prevê ações para prevenção de alterações visuais e avaliação da acuidade, em parceria com a escola^23^ . Verificou-se maior conhecimento dos usuários da ESF quanto a esses serviços prestados e maior disponibilidade destes, comparando-se com a UBSt.

O desempenho geral inadequado da integralidade dos serviços disponíveis pode indicar uma dificuldade real quanto ao oferecimento de uma variedade completa de ações e recursos diante das necessidades de saúde dos usuários^21 - 23^ . Para que haja integralidade dentro dos serviços de saúde, é necessário investir continuamente em recursos físicos, materiais e humanos^21 , 22^ . Constantemente, essa questão apresenta-se como um problema de difícil resolução dentro do serviço público, visto que muitos municípios apresentam instabilidade quanto ao provimento de recursos próprios e de financiamento estadual e federal, comprometendo a autonomia do sistema de saúde e o desenvolvimento das ações na APS^23^ .

Quanto à integralidade dos serviços prestados, o escore apresentou-se melhor para a ESF (≥ 6,6), indicando que os serviços prestados à criança se encontram deficitários no modelo de UBSt, corroborando resultados de estudo anterior^25^ .

Dessa forma, alguns tópicos importantes para a saúde da criança não estão sendo abordados consistentemente durante as consultas na UBSt. Dentre eles, destacam-se as questões sobre segurança no lar e formas de lidar com os problemas de comportamento da criança, que obtiveram escores muito baixos. Em estudo prévio, verificou-se que parte considerável da amostra avaliada afirmou não receber orientações sobre essas questões, mostrando a fragilidade dos serviços, já que se tratam de tecnologias leves e de baixo custo^4^ . Outros estudos obtiveram resultados semelhantes, evidenciando que o cuidado à criança está fragmentado, deixando de lado fatores mais amplos que interferem na saúde da criança^23 , 26^ .

Quanto à questão específica sobre crescimento e desenvolvimento da criança, observamos escores adequados para ambos os modelos. Essa questão é importante para o acompanhamento dos bebês de risco, visto que há um aumento da probabilidade da existência de doenças perinatais e infantis nessa população. O recém-nascido de risco deve ser acompanhado na APS até o segundo ano de vida, objetivando-se promoção da saúde, proteção e detecção precoce de alterações passíveis de modificação e que podem interferir na qualidade de vida da criança^27 , 28^ .

Uma hipótese para o alto escore detectado decorre do acompanhamento de crescimento da criança na APS, geralmente abordado de forma mais consistente^27^ , uma vez que está intimamente ligado a fatores biológicos, comumente priorizados^23^ . Quanto ao desenvolvimento, estudo anterior identificou que mais de 56% dos prontuários levantados na ESF e na UBSt não apresentaram registros referentes aos marcos de desenvolvimento^27^ , sugerindo que o acompanhamento pode não estar sendo efetivo. Essa possível fragilidade pode ficar mascarada pela abordagem simultânea desses dois aspectos em uma mesma questão^29^ .

Os resultados do presente estudo relacionados à integralidade (serviços disponíveis e serviços prestados) corroboram estudos prévios que mostraram que, mesmo na ESF, esse atributo ainda está aquém do esperado^23 , 30^ . Essa população, portanto, ainda se encontra em vulnerabilidade, o que pode acarretar prejuízos ao crescimento e desenvolvimento infantil, sendo necessário que maiores esforços sejam empreendidos por parte de profissionais e gestores dentro da organização e coordenação dos serviços da APS^23^ .

Quanto à orientação familiar e comunitária, apenas a ESF atingiu média adequada (orientação comunitária). Em relação ao cuidado com a criança, os escores apresentaram-se < 6,6, evidenciando que a aproximação entre serviços de saúde e famílias é insuficiente para ambos os modelos de gestão^3^ . Essa mesma tendência foi observada no conhecimento dos problemas de saúde da comunidade, sobretudo na UBSt, sugerindo que o atendimento das crianças pode estar desvinculado da realidade comunitária^3^ . Esses resultados podem também decorrer da falta de assimilação do modelo de atenção centrado na família e na comunidade^12^ . Espera-se que esses atributos sejam valorizados na ESF, o que pode justificar a melhor avaliação desse modelo.

Uma revisão sistemática analisou a avaliação dos atributos da APS feita por usuários de UBS por meio do PCATool. Em geral, o atributo acesso de primeiro contato apresentou baixos escores. A longitudinalidade foi bem avaliada na maioria dos estudos, assim como a integralidade, sendo esta superior na ESF em alguns deles, assim como a coordenação. Para os atributos orientação familiar e comunitária, o desempenho estava aquém do adequado, em quase todos os estudos; porém, com maiores escores na ESF^21^ .

Este estudo corrobora os resultados da revisão sistemática^21^ , observando-se que a ESF apresenta melhor desempenho geral. Quanto aos atributos, a UBSt mostrou baixos escores em todos, com exceção da coordenação dos sistemas de informação. A ESF apresentou baixos escores em acessibilidade, integralidade e orientação familiar, diferindo de alguns resultados da revisão.

Observamos baixos escores geral e essencial para ambos os modelos de gestão, mostrando que os responsáveis pelos bebês de risco avaliaram alguns atributos como insatisfatórios, destacando-se acessibilidade, integralidade e orientação familiar. Esses atributos são considerados fundamentais para a APS, e um desempenho aquém do esperado pode trazer consequências negativas para a qualidade e integralidade do cuidado ao bebê de risco, já que cabe ao serviço de APS criar mecanismos para atender às necessidades do indivíduo^19^ . Todas essas ações devem colocar a família como sujeito da atenção para integrar o cuidado do bebê de risco, em uma abordagem familiar e comunitária^15 , 21^ .

Cabe mencionar que o melhor desempenho da ESF, ainda que existam dificuldades presentes no cotidiano das UBS, reflete o investimento feito nesse modelo de gestão nos últimos anos para reestruturar a APS.
